# Therapeutic potential of tetrahydroxylated bile acids in reducing liver injury: Insights from the *Zfyve19^−/−^* mouse model

**DOI:** 10.1002/ped4.70036

**Published:** 2026-01-07

**Authors:** Li Wang, Yue Yu, Jiayan Feng, Yanan Zhang, Renxue Wang, Huiyu She, Teng Liu, Victor Ling, Jianshe Wang

**Affiliations:** ^1^ The Center for Pediatric Liver Diseases Children's Hospital of Fudan University, National Children's Medical Center Shanghai China; ^2^ Department of Pathology Children's Hospital of Fudan University, National Children's Medical Center Shanghai China; ^3^ BC Cancer Agency Vancouver British Columbia Canada; ^4^ Shanghai Key Laboratory of Birth Defect Shanghai China

**Keywords:** Cholestasis, Mouse, Portal fibrosis, Tetrahydroxylated bile acid, ZFYVE19

## Abstract

**Importance:**

The production of tetrahydroxylated bile acids (THBAs) is associated with better prognosis in some cholestatic patients as well as in multidrug resistance protein 2 knockout (*Mdr2^−/−^*) mice. However, it remains unclear whether this protective effect is specific to *Mdr2^−/−^* mice.

**Objective:**

To evaluate the effects of THBA (3α,6α,7α,12α‐Tetrahydroxy‐10β,13β‐pentanoic acid) in *Zfyve19^−/−^* mice, a newly developed mouse model characterized by cholestatic liver injury.

**Methods:**

THBA was administered to *Zfyve19^−/−^* mice challenged with alpha‐naphthyl isothiocyanate (ANIT). Serum biochemistry, liver histology and immunostaining, and quantitative PCR for hepatic expression of pro‐fibrotic, pro‐inflammatory, and bile acid metabolism‐related genes were performed and compared against ANIT‐treated wild‐type and *Zfyve19^−/−^* mice fed normal chow.

**Results:**

THBA administration reduced serum alanine aminotransferase (*P *< 0.001) and total bile acid levels (*P *< 0.001), decreased necrosis (*P* = 0.046), portal inflammation (*P *< 0.001), bile duct hyperplasia (*P* = 0.007), and portal fibrosis (*P* = 0.002) in liver histology, along with a significant reduction in hepatic expression of pro‐fibrotic genes (*Acta2*, *Col1a1*, *Tgfb1*, *Tgfb2*, and *Timp1*), as well as the pro‐inflammatory cytokines *Tnf* and macrophage chemokines (*Ccl2*, *Cxcl1*, *Cxcl9*, *Cxcl10*, and *Nos2*). Additionally, the mRNA expression of *Nr1h4* was profoundly upregulated, while key enzymes involved in bile acid synthesis were downregulated.

**Interpretation:**

THBA effectively alleviated cholestatic liver injury and fibrosis, and may represent a potential agent for the medical management of such diseases.

## Graphical abstract

THBA (3α,6α,7α,12α‐Tetrahydroxy‐10β,13β‐pentanoic acid) administration can alleviate cholestatic liver injury, hepatocellular necrosis, inflammatory response, bile duct hyperplasia, and portal fibrosis in the *Zfyve19^−/−^* mouse model. This evaluation encompasses various parameters, including serum biochemistry, liver histology, immunostaining, and quantitative polymerase chain reaction analysis of hepatic gene expression related to fibrosis, inflammation, and bile acid metabolism.

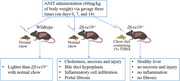

## INTRODUCTION

Bile acids, which serve as biological detergents, possess inherent cytotoxic properties and can potentially induce inflammatory stress in the liver. It is generally observed that hydrophobic bile acids exhibit higher toxicity than hydrophilic counterparts.^1^ Antagonizing bile acid toxicity is effective in the treatment of cholestatic liver diseases.^2^ Tetrahydroxylated bile acids (THBAs) are highly hydrophilic and typically undetectable in healthy individuals; however, they can be detected in cholestatic patients. Elevated THBA levels in the urine or plasma have been correlated with improved outcomes in infantile intrahepatic cholestasis patients.^1^, ^3^, ^4^ THBA also serves as a potential liver prognostic biomarker in patients with Alagille syndrome, with higher levels associated with better clinical outcomes.^5^ THBAs are also present at high levels in some cholestatic mouse models, such as bile salt export pump (*Bsep*/*Abcb11*) knockout (KO) mice.^6^, ^7^ Genetic defects in *ABCB11* cause severe progressive familial intrahepatic cholestasis type 2 in children; however, the *Abcb11^−/−^* mouse model is associated with a milder phenotype, lacking the development of progressive cholestasis.^6^, ^8^ High levels of THBA are associated with a mild phenotype in *Abcb11^−/−^* mice.^6^, ^7^ *Bsep* and multidrug resistance protein 2 (*Mdr2*) double KO mice (resulting in increased hydrophilicity of the bile pool) lack the liver pathology of their *Mdr2^−/−^* littermates, a mouse model of sclerosing cholangitis.^9^, ^10^ In addition, feeding with THBAs partially alleviated liver damage in *Mdr2^−/−^* mice, and the hepatic immune cell profile in THBA‐fed mice changed toward an anti‐inflammatory pattern.^9^, ^10^

Effective therapy is lacking for many patients with cholestatic liver injuries, such as those with primary sclerosing cholangitis, progressive familial intrahepatic cholestasis, and ciliopathy with hepatobiliary manifestations. Liver transplantation remains the only life‐extending treatment for patients with end‐stage liver disease. The association of increased THBA with good clinical outcomes and improved liver injury may indicate a potential therapeutic target. However, the therapeutic effects of THBA have only been studied in *Mdr2^−/−^* mice, a well‐established model of primary sclerosing cholangitis. It remains unclear whether this protective effect is specific to *Mdr2^−/−^* mice. We aimed to explore the protective effect of THBA against other hepatobiliary diseases to provide more evidence for its clinical use in the future.

Absence of zinc finger FYVE‐type containing 19 (ZFYVE19), a putative key regulator of the abscission checkpoint in cytokinesis,^11^ has recently been associated with a novel rare type of high γ‐glutamyl transpeptidase (GGT)‐progressive familial intrahepatic cholestasis, characterized by congenital hepatic fibrosis and sclerosing cholangiopathy (OMIM#619849).^12^, ^13^, ^14^ Recently, a *Zfyve19* KO mouse (*Zfyve19^−/−^*) was shown to exhibit cholestasis, portal inflammation, bile duct hyperplasia, and portal fibrosis when challenged with the hepatobiliary toxin alpha‐naphthyl isothiocyanate (ANIT).^15^, ^16^ These observed features correspond to the central morphological characteristics associated with ZFYVE19 deficiency,^12^ rendering the mouse model suitable for preclinical studies of THBA. Further studies showed that the absence of *ZFYVE19/Zfyve19* expression causes failure of cell division, with ciliary and centriolar abnormalities and cell death, thus initiating the process of biliary fibrosis, indicating that ZFYVE19 deficiency may be a ciliopathy with hepatobiliary manifestations.^15^

In this study, we investigated the effect of THBA administration in a newly established gene‐deficient mouse model, thereby shedding new light on the potential role of THBA as a novel therapeutic agent for ciliopathy with hepatobiliary disease.

## METHODS

### Ethics approval

All animal protocols were approved by the Committee of the Care and Use of Laboratory Animals at Children's Hospital of Fudan University (permit number: No. (2022) 103/104) in accordance with the guidelines of the Institutional Animal Care and Use Committee (IACUC).

### Animals and ANIT‐induced liver disease

*Zfyve19* ^−/−^ mice in the C57BL/6N background were generated by deleting exons 3–6 of *Zfyve19* (NM_028054.3) using the CRISPR/Cas9 system in collaboration with Cyagen Biosciences, as previously described.^15^ Deletion was confirmed at the DNA, mRNA, and protein levels. Human ZFYVE19 deficiency manifestations, including elevated serum liver injury markers, increased bile duct profiles, portal inflammation, and fibrosis, were consistently induced in male *Zfyve19^−/−^* mice (6–8 weeks) by administering ANIT (60 mg/kg of body weight) via gavage three times (on days 0, 7, and 14).^15^ All mice were housed in a specific pathogen‐free and temperature‐controlled (20–22°C) environment with a 12 h light‐dark cycle and free access to mouse chow and drinking water. Animal experiments were performed in age‐matched littermates of male wild‐type (WT) and *Zfyve19^−/−^* mice (6–8 weeks), which were orally administered a dose (60 mg/kg of body weight) of ANIT (Sigma‐Aldrich, N‐4525) in olive oil three times (on days 0, 7, and 14).^15^, ^16^

### THBA administration

**FIGURE 1:**
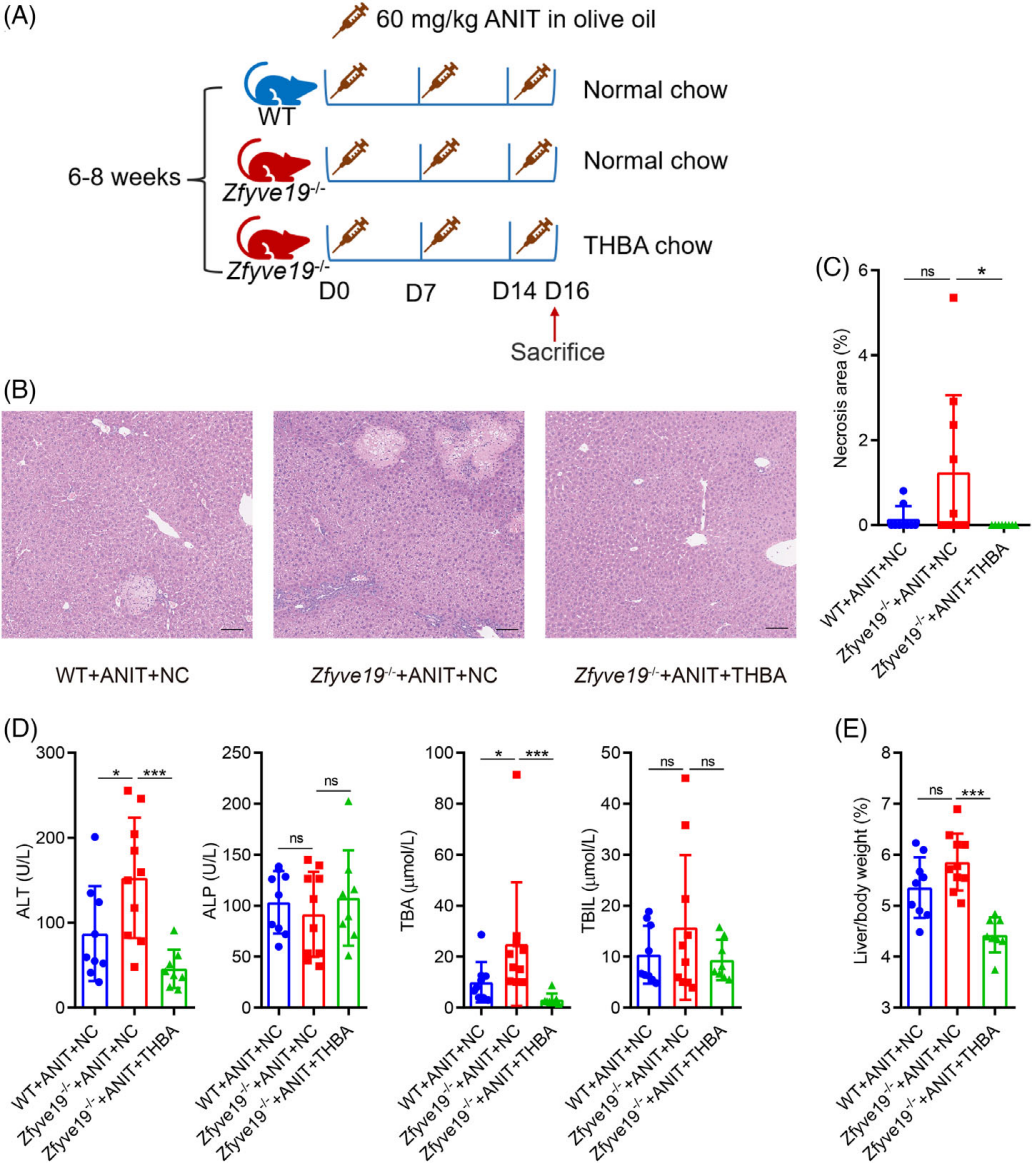
THBA reduced cholestatic liver injury in ANIT‐treated *Zfyve19^−^^/^^−^* mice. (A) Schematic experimental outline (*n* = 8−10 in each group). (B) Representative H&E images in each group (bar = 100 µm). (C) Quantification of necrotic areas. (D) Serum level of ALT, ALP, TBA, and TBIL. (E) Liver weight to body weight ratio. Data are shown as mean ± standard deviation. ^*^ *P* < 0.05; ^***^ *P* < 0.001. ALP, alkaline phosphatase; ALT, alanine aminotransferase; ANIT, alpha‐naphthyl isothiocyanate; NC, normal chow; ns, not significant; TBA, total bile acid; TBIL, total bilirubin; THBA, tetrahydroxylated bile acid; WT, wild‐type.

*Zfyve19* ^−/−^ mice did not show differences from WT littermates.^15^ To investigate the therapeutic effects of THBA, three groups of mice were challenged with ANIT. A schematic of the experimental outline is shown in Figure 1A. The THBA group consisted of *Zfyve19^−/−^* mice fed a chow diet containing 1% THBA from the day of initial ANIT gavage. Both control groups were fed normal chow throughout: control group 1 consisted of *Zfyve19^−/−^* mice, while control group 2 consisted of WT mice. All mice were gavaged with ANIT weekly from 6–8 weeks of age for three times and sacrificed 36–48 h after the third ANIT gavage, following a 2–4 hour fasting period, and samples were collected for further analysis. The THBA (3α,6α,7α,12α‐Tetrahydroxy‐10β,13β‐pentanoic acid) powder was provided by Victor Ling from BC Cancer Research Centre, and the 1% THBA diet was prepared by XIETONGSHENGWU (Jiangsu, China).

### Serum biochemistry

The blood samples were centrifuged at a relative centrifugal force of 8000 g for 10 min to obtain serum, which was then stored at −80°C until further analysis. Serum samples were analyzed for alanine aminotransferase (ALT), alkaline phosphatase (ALP), total bile acid (TBA), and total bilirubin (TBIL) levels using commercial kits (Rayto Life and Analytical Sciences Co., Ltd., Shenzhen, China; ALT: S03030; AP: S03038; TBA: S03074; Changchun Huili Biotech Co., Ltd., Changchun, China; TB: C120).

### Histology

Liver tissue from the center of the largest lobule was fixed in 4% paraformaldehyde and embedded in paraffin. Sections were cut to a thickness of 4 µm. Hematoxylin and eosin (H&E), Sirius Red, and immunohistochemistry (IHC) staining for cytokeratin‐19 (CK19, ab52625, Abcam), CD45 (ab208022, Abcam), and F4/80 (MCA497R, Bio‐Rad) were performed on liver tissues according to standard protocols. All the slides were imaged using a Hamamatsu NanoZoomer (Hamamatsu, Japan). Quantification of necrosis, Sirius Red, CK19, and F4/80 positive areas, as well as CD45^+^ cells, was performed using ImageJ software by analyzing 8 random and non‐overlapping fields per mouse at the same magnification. Representative images from each group are shown.

### RNA analysis

Total RNA was extracted from mouse liver tissues using the Direct‐zol RNA Miniprep kit (R2050, Zymo Research) and reverse transcribed using the PrimeScript RT reagent Kit with gDNA Eraser (RR047A, Takara). For real‐time quantitative polymerase chain reaction (qPCR) analysis, selected primers (Table S1) were synthesized by Biosune (Shanghai, China), and the experiment was performed on a QuantStudio 3 Real‐Time PCR System (Applied Biosystems) using TB Green^®^ Premix Ex Taq (RR420A, Takara). Data were normalized to *Gapdh* as a housekeeping gene.

### Statistical analysis

Data were expressed as mean ± standard deviation of at least 8–10 animals/three independent experiments. Student's unpaired *t‐*test or Mann‐Whitney *U* test was used to determine differences between groups, as appropriate, using SPSS 23 and GraphPad Prism software (version 8.0). A *P* value of less than 0.05 was considered statistically significant.

## RESULTS

### THBA reduced cholestatic liver injury in ANIT‐treated *Zfyve19* ^−/−^ mice

As shown in Figure 1B, C, compared to ANIT‐treated WT mice, ANIT‐treated *Zfyve19^−/−^* mice without THBA feeding exhibited more severe cholestatic liver injury, consistent with a previous study.^15^, ^16^ THBA feeding reduced cholestatic liver injury, as shown by H&E staining (Figure 1B). Among the ANIT‐treated *Zfyve19^−/−^* mice fed normal chow, five out of 10 exhibited extensive hepatocellular necrosis. In contrast, none of the ANIT‐treated *Zfyve19^−/−^* mice fed THBA showed signs of hepatocellular necrosis. This suggests that THBA may reverse hepatocellular necrosis in ANIT‐treated *Zfyve19^−/−^* mice (Figure 1C). Consistent with these histopathological improvements, serum ALT level (152.8 ± 70.9 vs. 45.8 ± 22.6 U/L, *P *< 0.001) as a marker of hepatocellular injury and TBA (24.9 ± 24.2 vs. 3.2 ± 2.3 µmol/L, *P* < 0.001) as a marker of cholestasis were significantly reduced, though other serum markers of cholestasis (ALP and TBIL) showed no significant differences by THBA treatment (Figure 1D). Correspondingly, the liver/body weight ratio also remarkably decreased in *Zfyve19* ^−/−^ mice after THBA treatment (Figure 1E), indicating the beneficial effects of THBA on liver injury.

### THBA reduced bile duct hyperplasia in ANIT‐treated *Zfyve19^−/−^* mice

**FIGURE 2:**
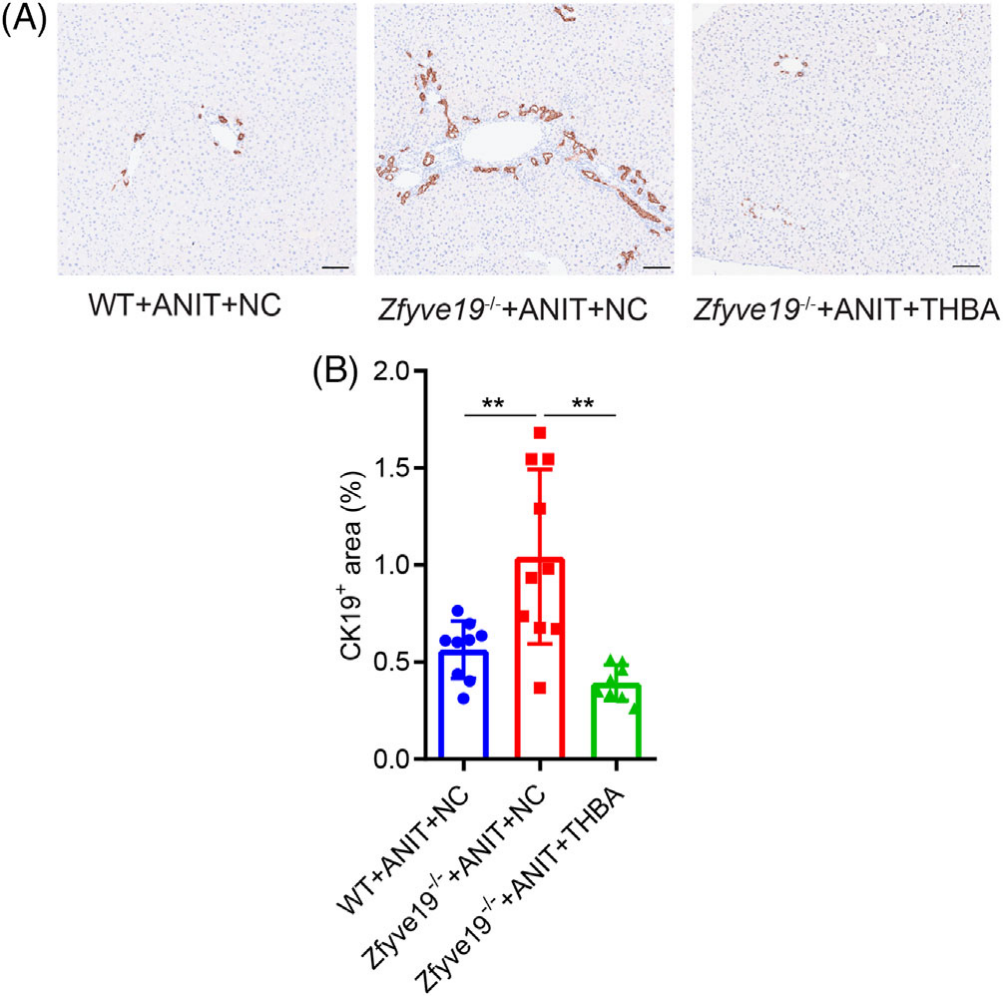
THBA reduced bile duct hyperplasia in ANIT‐treated *Zfyve19^−^^/^^−^* mice. (A) Representative immunohistochemical images of CK19 in each group (bar = 100 µm). (B) Quantification of CK19. Data are shown as mean ± standard deviation (*n* = 8−10 per group). ^**^ *P* < 0.01. ANIT, alpha‐naphthyl isothiocyanate; CK19, cytokeratin‐19; NC, normal chow; THBA, tetrahydroxylated bile acid; WT, wild‐type.

Quantification of the CK19‐positive area determined by CK19 IHC staining was used as an indicator of the degree of bile duct hyperplasia (Figure 2A). CK19 positive area (0.56% ± 0.15% vs. 1.04% ± 0.45%, *P * =  0.007) was increased in ANIT‐treated *Zfyve19* ^−/−^ mice compared to that in WT mice, while it was significantly reduced (1.04% ± 0.45% vs. 0.39% ± 0.09%, *P * = 0.001) in ANIT‐treated *Zfyve19* ^−/−^ mice when administered with THBA (Figure 2A, B). These results show that THBA treatment can reduce or prevent ductular reactions in this mouse model.

### THBA decreased portal fibrosis in ANIT‐treated *Zfyve19^−/−^* mice

**FIGURE 3:**
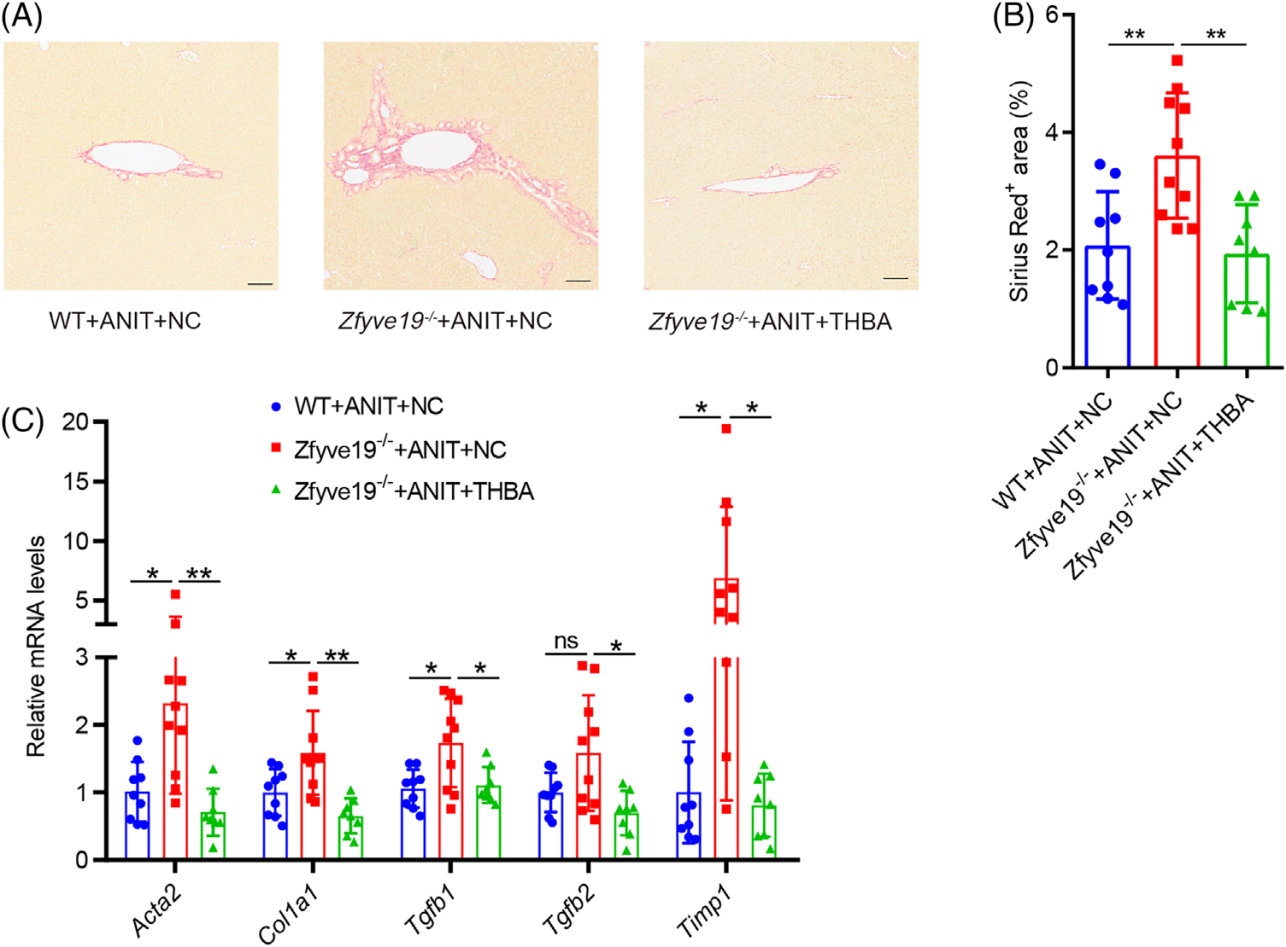
THBA decreased portal fibrosis in ANIT‐treated *Zfyve19^−^^/^^−^* mice. (A) Representative Sirius Red staining in each group (bar = 100 µm). (B) Quantification of fibrotic area. (C) Relative hepatic mRNA expression of *Acta2*, *Col1a1*, *Tgfb1*, *Tgfb2*, and *Timp1*. Data are shown as mean ± standard deviation (*n* = 8−10 per group). ^*^ *P* < 0.05; ^**^ *P* < 0.01. ANIT, alpha‐naphthyl isothiocyanate; NC, normal chow; ns, not significant; THBA, tetrahydroxylated bile acid; WT, wild‐type.

Sirius Red staining showed that portal expansion and fibrosis were more prominent in ANIT‐treated *Zfyve19^−/−^* mice than in WT mice (Figure 3A), as previously reported.^15^, ^16^ THBA feeding significantly reduced the fibrotic area in ANIT‐treated *Zfyve19^−/−^* mice (Figure 3A, B). Consistent with the Sirius Red staining results, the administration of THBA significantly decreased the mRNA expression of genes involved in fibrogenesis, including *Acta2* (*P *= 0.007), *Col1a1* (*P *= 0.003), *Tgfb1* (*P *= 0.023), *Tgfb2* (*P *= 0.011), and *Timp1* (*P *= 0.045) (Figure 3C). These results suggest that THBA effectively alleviates portal fibrosis.

### THBA exhibited an anti‐inflammatory effect in ANIT‐treated *Zfyve19^−/−^* mice

**FIGURE 4:**
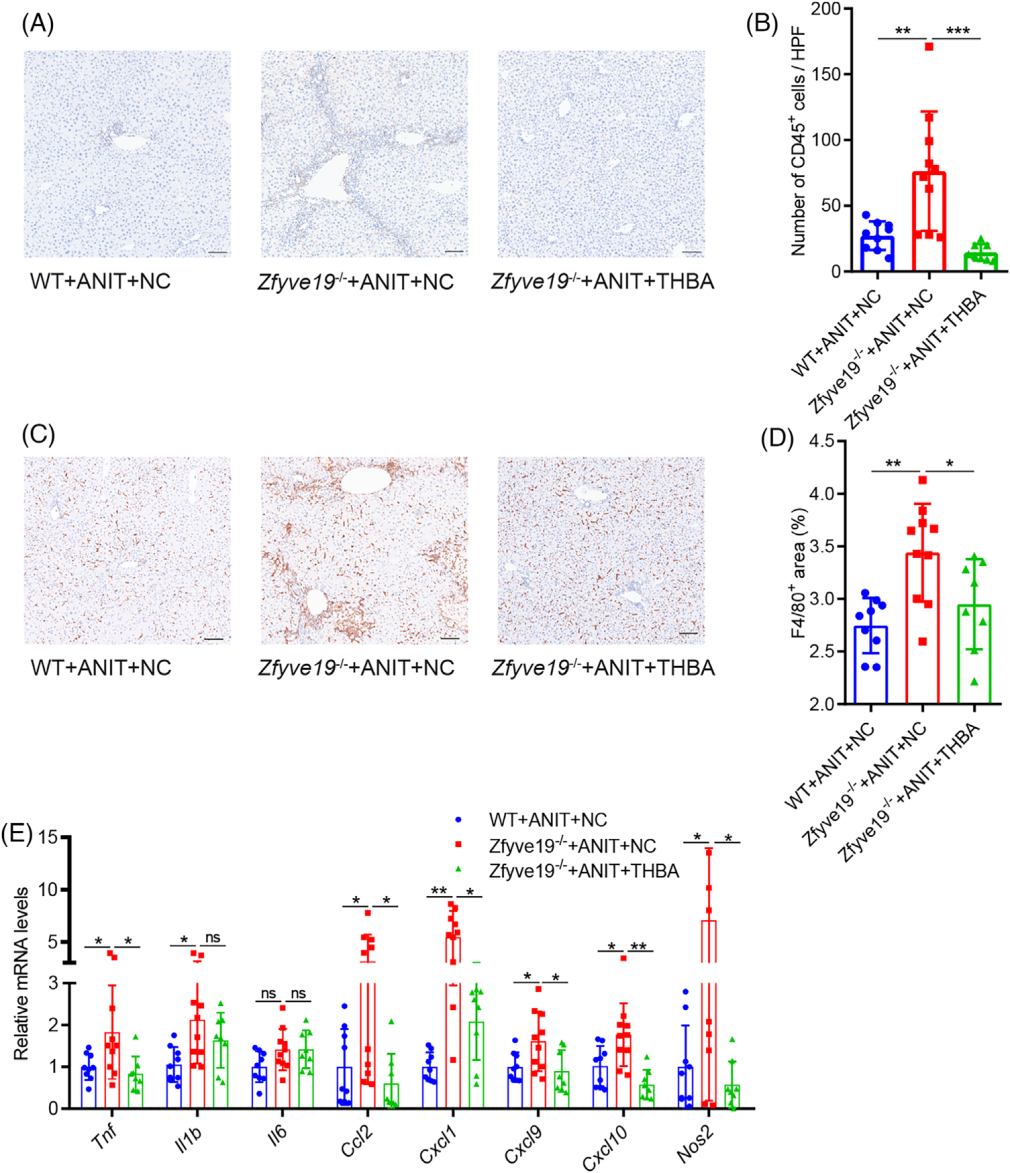
THBA decreased hepatic inflammation in ANIT‐treated *Zfyve19^−^^/^^−^* mice. (A) Representative immunohistochemical images of CD45 in each group (bar = 100 µm). (B) Quantification of CD45^+^ cells. (C) Representative immunohistochemical images of F4/80 in each group (bar = 100 µm). (D) Quantification of F4/80. (E) Relative hepatic mRNA expression of pro‐inflammatory cytokine *Tnf*, *Il1b*, and *Il6*, and the macrophage chemokines *Ccl2*, *Cxcl1*, *Cxcl9*, *Cxcl10*, and *Nos2*. Data are shown as mean ± standard deviation (*n* = 8−10 per group). ^*^ *P* < 0.05; ^**^ *P* < 0.01; ^***^ *P* < 0.001. ANIT, alpha‐naphthyl isothiocyanate; NC, normal chow; ns, not significant; THBA, tetrahydroxylated bile acid; WT, wild‐type.

To evaluate the potential effect of THBA on inflammatory cell infiltration, liver tissues were stained with antibodies specific for CD45 (leukocytes) and F4/80 (macrophages). As shown in Figure 4A–D, the number of CD45 and F4/80 positive inflammatory cells was significantly increased in ANIT‐treated *Zfyve19^−/−^* mice, with accumulation primarily localized to the portal area. However, THBA administration markedly reduced the infiltration of these inflammatory cells, restoring the distribution pattern resembling that observed in WT mice. Consistent with the results related to inflammatory cell infiltration, the mRNA expression of pro‐inflammatory cytokines and macrophage chemokines, including *Tnf* (*P * = 0.021), *Ccl2* (*P * = 0.019), *Cxcl1* (*P * = 0.014), *Cxcl9* (*P * = 0.011), *Cxcl10* (*P * = 0.006), and *Nos2* (*P * = 0.018), was significantly reduced after THBA feeding (Figure 4E). Although the changes in the mRNA expression of *Il6* and *Il1b* were not significant, a downward trend was noticeable. These results suggested that THBA treatment may have anti‐inflammatory effects.

### Effect of THBA on bile acid homeostasis in ANIT‐treated *Zfyve19^−/−^* mice

**FIGURE 5:**
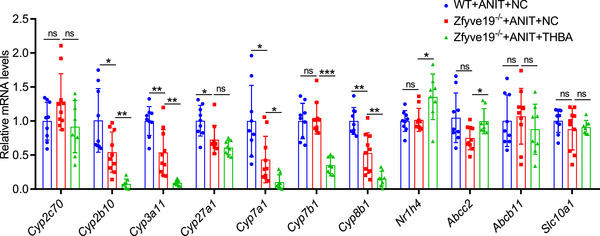
Effect of THBA on bile acid homeostasis in ANIT‐treated *Zfyve19^−^^/−^* mice. Relative hepatic mRNA expression of *Cyp2c70*, *Cyp2b10*, *Cyp3a11*, *Cyp27a1*, *Cyp7a1*, *Cyp7b1*, *Cyp8b1*, *Nr1h4*, *Abcc2*, *Abcb11*, and *Slc10a1*. Data are shown as mean ± standard deviation (*n* = 8−10 per group). ^*^ *P* < 0.05; ^**^ *P* < 0.01; ^***^ *P* < 0.001. ANIT, alpha‐naphthyl isothiocyanate; NC, normal chow; ns, not significant; THBA, tetrahydroxylated bile acid; WT, wild‐type.

The effect of THBA supplementation on the mRNA expression of enzymes involved in bile acid metabolism is shown in Figure 5. The hepatic mRNA expression of enzymes involved in the classic pathway of bile acid synthesis, including *Cyp7a1* and *Cyp7b1*, was significantly reduced after THBA administration. Additionally, the mRNA levels of genes associated with the alternative bile acid synthesis pathway, including *Cyp8b1* and *Cyp27a1*, were reduced; however, only the reduction in *Cyp8b1* was statistically significant. The mRNA expression of the bile acid‐detoxifying enzymes *Cyp3a11* and *Cyp2b10* was also reduced. The mRNA expression of *Cyp2c70*, the principal enzyme involved in the generation of hydrophilic muricholic acids in mice, was also decreased in THBA‐fed mice, but the difference was not statistically significant. In addition, the expression of *Nr1h4* (a nuclear receptor of bile acids, *P *= 0.046) and *Abcc2* (involved in bile acid efflux, *P *= 0.032) was significantly increased in THBA‐fed mice. However, the hepatic mRNA levels of *Abcb11* (responsible for bile acid transport from hepatocytes to the canalicular region) and *Slc10a1* (a solute carrier family member facilitating hepatic bile acid uptake) did not differ in THBA‐fed mice. These results suggest that THBA feeding may decrease hepatocellular bile acid load and bile acid toxicity.

## DISCUSSION

Therapeutic options for chronic cholestatic liver disease, particularly hereditary cholestasis, are limited and often ineffective. End‐stage liver disease develops in 40%–50% of reported patients with ZFYVE19 deficiency, often necessitating liver transplantation.^12^ Therefore, there is an urgent need to explore new treatment strategies to improve patient outcomes. A crucial consideration in the development and evaluation of treatments for human cholestasis is the reduction in both bile acid pool size and hydrophobicity.^17^ Our study demonstrates that THBA, a highly hydrophilic bile acid, can alleviate cholestatic liver injury in the *Zfyve19^−/−^* mouse model. THBA treatment resulted in a reduction in hepatocellular necrotic areas, inflammatory responses, bile duct hyperplasia, and portal fibrosis, as evidenced by liver histology and reduced serum liver enzyme and TBA levels. We also observed a decrease in the mRNA levels of pro‐fibrotic and pro‐inflammatory mediators in *Zfyve19^−/−^* mice administered 1% THBA. Taken together, our findings indicate that THBA may have potential therapeutic implications in cholestatic liver injury.

THBA is more hydrophilic and less cytotoxic than usual bile acids.^1^ Mice lacking Bsep, the primary bile acid transporter in the liver, do not exhibit severe liver damage, typically observed in individuals with Bsep deficiency. Moreover, these mice can protect themselves from cholestatic liver disease induced by bile duct ligation. This protective effect may be attributed to the ability of these mice to detoxify hydrophobic bile acids through the production of THBA.^6^, ^7^ Deficiency of Mdr2 in mice is commonly used as a model of sclerosing cholangitis. These mice have been observed to develop pericholangitis, ductular proliferation, and onion skin‐type periductal fibrosis spontaneously.^18^, ^19^ In contrast, *Mdr2^−/−^ Bsep^−/−^* double KO mice displayed a very mild phenotype and, similar to *Bsep^−/−^* mice, produced higher levels of THBA. Additionally, feeding *Mdr2^−/−^* mice with THBA partially alleviates liver damage.^9^, ^10^

In our *Zfyve19^−/−^* mice, the second mouse model, treated with THBA, also demonstrated significant therapeutic benefits. The observed improvements in the histological and biochemical features of liver injury in the *Zfyve19^−/−^* mouse model may be attributed to a more hydrophilic bile acid pool, decreased synthesis of bile acids, and enhanced hepatic bile acid clearance following THBA supplementation. First, the significantly decreased mRNA expression of the detoxifying enzymes *Cyp2b10* and *Cyp3a11*, whose activities enhance bile salt hydrophilicity, in THBA‐fed *Zfyve19^−/−^* mice may imply less toxic bile when THBA is administered to *Zfyve19^−/−^* mice, thereby minimizing liver damage. Intriguingly, the expression levels of *Cyp2b10* and *Cyp3a11* were also lower in *Zfyve19* ^−/−^ mice than in WT mice fed normal chow. This finding implies that *Zfyve19^−/−^* mice are unable to activate the detoxification enzyme system to compensate for cholestasis, underscoring the importance of exogenous supplementation with hydrophilic bile acids in the treatment of such diseases. Second, our results indicated that THBA feeding upregulated the mRNA expression of *Nr1h4* while downregulating the expression of key bile acid synthesis enzymes, *Cyp7a1*, *Cyp7b1*, and *Cyp8b1*. This suggests the possibility of a further decrease in bile acid synthesis in THBA‐fed *Zfyve19^−/−^* mice. At the same time, the expression levels of *Cyp7a1* and *Cyp8b1*, as well as the alternative bile acid synthesis pathway‐related gene *Cyp27A1*, were also decreased in *Zfyve19* ^−/−^ mice compared to WT mice fed normal chow. This likely reflects negative feedback due to the accumulation of bile acids in the liver, as indicated by the elevated serum total bile acids in *Zfyve19^−/−^* mice fed normal chow. These findings suggest that THBA may more effectively suppress bile acid synthesis without increasing the overall bile acid load, as evidenced by the lower hepatic expression of bile acid synthesis enzymes and lower serum bile acid levels in THBA‐fed mice. Additionally, THBA‐fed *Zfyve19^−/−^* mice showed substantially higher expression of the ABC transporter *Abcc2*, which appears to be responsible for the transport of certain bile acids, including THBA, from hepatocytes into the canaliculus.^20^, ^21^ However, the mRNA expression of *Abcb11* and *Slc10a1* remained unchanged. These results suggest that THBA is not a primary substrate for the two major bile acid transporters examined, implying that its beneficial effects are not mediated through direct transport by these proteins. Collectively, THBA feeding may potentially safeguard *Zfyve19^−/−^* mice from the development of cholestatic liver injury by promoting a more hydrophilic bile acid pool, reducing bile acid synthesis, and partially enhancing bile acid extransport.

The therapeutic effects of THBA may also result from its ability to alleviate or prevent liver inflammation and fibrosis induced by toxic bile acids. Our previous work showed that deficiency of ZFYVE19 causes failure of cell division and increased cell death, which may trigger the release of chemokines and activate macrophages via transforming growth factor beta (TGF‐β) signaling, ultimately leading to biliary injury and fibrosis.^15^ Notably, upregulation of the TGF‐β signaling pathway is commonly associated with fibrosis and macrophage activation.^22^, ^23^, ^24^, ^25^ In recent years, it has been increasingly recognized that inflammatory macrophages play an important role in driving portal fibrosis progression, and inhibiting inflammatory signaling through genetic or pharmacological methods can improve periportal fibrosis.^26^, ^27^, ^28^, ^29^, ^30^, ^31^ In this study, *Zfyve19^−/−^* mice were fed THBA from the onset of ANIT administration, which may prevent abnormal cell division, reduce cell death, and thereby inhibit aberrant bile duct proliferation, and alleviate bile duct injury and fibrosis. Liver histology revealed that portal fibrosis, bile duct hyperplasia, and hepatic inflammation were not evident in THBA‐fed *Zfyve19^−/−^* mice. In agreement with the histological observations, whole‐liver qPCR analysis revealed a notable decrease in the expression of *Tgfb1* and *Tgfb2*, which are involved in the activation of the TGF‐β signaling pathway and fibrogenesis‐related genes, such as *Acta2*, *Col1a1*, and *Timp1*, in mice fed THBA. In a previous study, RNA sequencing of human cirrhotic livers revealed that *CCL2* is one of the key chemokines upregulated by scar‐associated macrophages after differentiation from monocytes and is important for fibrotic niche formation.^30^, ^32^ In this study, we observed a significant reduction in the macrophage chemokine *Ccl2*, along with other macrophage chemokines (*Cxcl1*, *Cxcl9*, *Cxcl10*, and *Nos2*), and the pro‐inflammatory cytokine *Tnf* in *Zfyve19^−/−^* mice fed THBA. This suggests that THBA may modulate the activation of the TGF‐β signaling pathway and macrophages, thus attenuating or preventing liver inflammation, fibrosis progression, bile duct hyperplasia, and cholestatic liver injury.

Although the animal care and ANIT administration in the present study were identical to those used in our previous work,^15^ the extent of liver injury and cholestasis in the present model, as assessed by serum biochemical markers, appeared milder. This discrepancy may arise from the variability between animal batches. However, all the mice used in this study were derived from the same batch, and the histopathological features were consistent with our previous findings, confirming the suitability of the model for evaluating the therapeutic efficacy of THBA. The exclusive use of male mice represents a limitation as it may reduce the translational value of the study. Additionally, while total serum bile acid levels were measured, the absence of bile acid profiling limited mechanistic insight into specific alterations in bile acid composition. Future studies, including comprehensive bile acid profiling, are warranted to clarify the roles of individual bile acids in mediating the therapeutic effects of THBA.

In conclusion, this study demonstrated that THBA administration can reduce serum biomarkers of cholestatic liver injury, hepatocellular necrosis, inflammatory response, bile duct hyperplasia, and portal fibrosis in the *Zfyve19^−/−^* mouse model. Our results suggest that THBA administration may open new avenues for the pharmacological treatment of cholestatic liver diseases. However, further studies are warranted to fully understand the implications of bile acid regulation and the potential long‐term effects of THBA.

## CONFLICT OF INTEREST

The authors declare no conflict of interest.

## Supporting information



Supporting Information
