# Supplementary material for: Therapeutic potential of tetrahydroxylated bile acids in reducing liver injury: Insights from the Zfyve19−/− mouse model
Source: Pediatr Investig. 2026 Jan 7;10(1):60–9. doi: 10.1002/ped4.70036 (PMC12921634; doi:10.1002/ped4.70036)
Supplement: Supplementary file 1 — Supporting Information [file PED4-10-60-s001.pdf]

Supplementary Material for

Therapeutic potential of tetrahydroxylated bile acids in reducing liver injury: insights from the *Zfyve19*$^{-/-}$ mouse model

Li Wang, Yue Yu, Jiayan Feng, Yanan Zhang, Renxue Wang, Huiyu She, Teng Liu, Victor Ling, Jianshe Wang

Table S1 Primers for qPCR

Gene	Forward primer (5' to 3')	Reverse primer (5' to 3')
Acta2	AAACAGGAATACGACGAAG	CAGGAATGATTTGGAAAGGA
Col1a1	ACGTGGAAACCCGAGGTATG	TTGGGTCCCTCGACTCCTAC
Timp1	CGAGACCACCTTATACCAGCG	ATGACTGGGGTGTAGGCGTA
Tgfb1	AGGGCTACCATGCCAACTTC	CCACGTAGTAGACGATGGC
Tgfb2	TCGACATGGATCAGTTTATGCG	CCCTGGTACTGTTGTAGATGGA
Ccl2	TAAAAACCTGGATCGGAACCAAA	GCATTAGCTTCAGATTTACGGGT
Cxcl1	ACTGCACCCAAACCGAAGTC	TGGGGACACCTTTTAGCATCTT
Cxcl10	CCAAGTGCTGCCGTCATTTTC	GGCTCGCAGGGATGATTTCAA
Cxcl9	TGGAGCAGTGTGGAGTTCG	GTAGTGGATCGTGCCTCGG
Nos2	ACATCGACCCGTCCACAGTAT	CAGAGGGGTAGGCTTGTCTC
Tnf	GGAACTGGCAGAAGAGGCACTC	GCAGGAATGAGAAGAGGCTGAGAC
Il1b	CCCAACTGGTACATCAGCACCTC	GACACGGATTCCATGGTGAAGTC
Il6	TAGTCCTTCCTACCCCAATTTCC	TTGGTCCTTAGCCACTCCTTC
Cyp2b10	TTAGTGGAGGAACTGCGGAAA	CGCAAGAACTGACGGTCTG
Cyp3a11	CTTGGTGCTCCTCTACCGATATG	TGGGTCTGTGACAGCAAGGA
Cyp2c70	CTGTGCAGTGCCTACCCTATT	TCCAGAGATTTTTGATGCCTGTT
Cyp7b1	CTATGGAAGCCCTGCGTGAC	GAGCACAGCCTCAGAACCTC
Cyp7a1	GGTGGTGAGAGCTTGAAAATGA	GTGGTTCTTGGAGGTGCCTTT
Cyp27a1	GGACAACCTCCTTTGGGACTT	ATTGGGTACTTGCCCTCCTG
Cyp8b1	TGCAAAAGAACTGGTGCTCAA	CGAACCTTTAGGCCCTAGCAT
Nr1h4	CAGGGTTTCAGACACTGGATCACG	AGGTCTGCATGACCGGCAGGAAGT
Abcc2	GCAACTCTACTTTTTGGAATCTCT	CCAAGAGCCAAAGAAAGCCC
Abcb11	TGGATCAACAGCTCCTTCAA	ACACCAACTCCTGCGTAGA
Slc10a1	GGTGCCCTACAAAGGCATTA	ACAGCCACAGAGAGGGAGAA
Gapdh	AGGTCGGTGTGAACGGATTTG	TGTAGACCATGTAGTTGAGGTC
